# PDMS Based Hybrid Sol-Gel Materials for Sensing Applications in Alkaline Environments: Synthesis and Characterization

**DOI:** 10.3390/polym12020371

**Published:** 2020-02-07

**Authors:** Rui P. C. L. Sousa, Bárbara Ferreira, Miguel Azenha, Susana P. G. Costa, Carlos J. R. Silva, Rita B. Figueira

**Affiliations:** 1 Centro de Química, Campus de Gualtar, Universidade do Minho, 4710-057 Braga, Portugal; barbarafoferreira.730@gmail.com (B.F.); spc@quimica.uminho.pt (S.P.G.C.); csilva@quimica.uminho.pt (C.J.R.S.); 2 ISISE, Departamento de Engenharia Civil, Escola de Engenharia, Campus de Azurém, Universidade do Minho, 4800-058 Guimarães, Portugal; miguel.azenha@civil.uminho.pt

**Keywords:** sol-gel, PDMS, pH, hybrid, phenolphthalein

## Abstract

Nowadays, concrete degradation is a major problem in the civil engineering field. Concrete carbonation, one of the main sources of structures’ degradation, causes concrete’s pH to decrease; hence, enabling the necessary conditions for corrosion reinforcement. An accurate, non-destructive sensor able to monitor the pH decrease resistant to concrete conditions is envisaged by many researchers. Optical fibre sensors (OFS) are generally used for concrete applications due to their high sensitivity and resistance to external interferences. Organic-inorganic hybrid (OIH) films, for potential functionalization of OFS to be applied in concrete structures, were developed. Polydimethylsiloxane (PDMS) based sol-gel materials were synthesized by the formation of an amino alcohol precursor followed by hydrolysis and condensation. Different ratios between PDMS and (3-aminopropyl)triethoxysilane (3-APTES) were studied. The synthesized OIH films were characterized by Fourier-transformed infrared spectroscopy (FTIR), UV–Vis spectroscopy, electrochemical impedance spectroscopy (EIS) and thermogravimetric analysis (TGA). The OIH films were doped with phenolphthalein (Phph), a pH indicator, and were characterized by UV–Vis and EIS. FTIR characterization showed that the reaction between both precursors, the hydrolysis and the condensation reactions occurred successfully. UV–Vis characterization confirmed the presence of Phph embedded in the OIH matrices. Dielectric and thermal properties of the materials showed promising properties for application in contact with a high alkaline environment.

## 1. Introduction

The sol-gel process is a synthetic method that allows producing organic-inorganic hybrid (OIH) materials [1,2]. These materials are also known as organically modified silicates (ORMOSILs), and have a wide range of applications [3,4,5,6,7,8,9,10,11,12]. Generically, this method consists of the hydrolysis and condensation of an alkoxide to form a polymeric matrix [13,14,15,16]. The precursors and the synthesis conditions can be tuned, allowing one to obtain a product with suitable physicochemical properties according to the required application. Furthermore, OIH sol-gel materials can be doped with several species, such as corrosion inhibitors [17], electrolytes (to produce high conductivity films) [18], pharmaceutical drugs or other biomolecules [19]. These materials can also be doped with chemosensors, allowing them to obtain a polymeric matrix with sensing abilities [20,21,22]. A chemosensor is a molecule that is sensitive to the presence of a certain analyte and provides a detectable change in a signal, transducing a chemical signal into an action potential [23]; pH indicators are one of the main examples of this class of chemosensors, since these molecules provide a signal change with a concentration variation in H^+^; pH indicators such as cresol red, bromophenol blue and fluorescein, among others, have already been successfully entrapped into a polymeric sol-gel matrix [24]. The most sol-gel precursors used in these cases are tetramethyl orthosilicate (TMOS) and tetraethyl orthosilicate (TEOS). The literature reports also entrapment of pH indicators, such as fluorescein isothiocyanate and hydroxypyrenetrisulfonic acid [25,26], in films based on polydimethylsiloxanes (PDMS). However, these studies only covered neutral pH values. PDMS is one of the most used precursors in sol-gel synthesis due to the presence of different functional groups such as amine [27], hydroxyl [28] and epoxy [29]. PDMS based precursors are commonly viscous liquids, and several applications such as coatings [30], sponges [31] or biomedical devices [32] have been reported. The different PDMS functional groups available allow the possibility to synthesize OIH gel matrices by reacting with a cross-linker, such as a silane [33,34,35], followed by hydrolysis and condensation. These precursors show several advantages when compared to other sol-gel precursors (e.g., TEOS or TMOS, which produce fully inorganic materials), particularly in properties such as elasticity, transparency and biocompatibility [36]. These properties allowing one to obtain OIH films suitable for optical fibre sensing applications such as the ones reported by Gao and Wang [37,38].

Concrete structures are designed to have high durability (design lifetimes typically within 50 to 100 years) with low maintenance costs. Therefore, concrete can be regarded as a stable material, with the typical internal pH at the pore network having, usually, very high values (12.5–13.5). However, due to the combination of several factors, the degradation of concrete and reinforced concrete structures (RCS) may take place due to corrosion or chemical reactions such as the alkali–silica reaction [39,40]. Moreover, the porous structure of the concrete does not provide a perfect physical barrier. This lack of imperviousness allows the progressive ingress of aggressive species at the steel/concrete interface, causing the rupture of the passivation film. The most common causes for RCS corrosion are the ingress of chloride ions, the reaction of atmospheric CO_2_ with the constituents of concrete and the combination of these two processes. Carbonation of concrete occurs due to the chemical reaction of atmospheric CO_2_ with the alkaline components present in the concrete pore solution, forming calcite (CaCO_3_). This reaction leads to a decrease in the pH values of the interstitial concrete pore solution [41]. This pH decrease, generally from values above 12.5 to values between 9 and 6, compromises the concrete’s protective function [42]. Therefore, monitoring concrete and RCS is a crucial activity, not only economically but also for human safety; pH monitoring in concrete has been approached by the scientific community already. For instance, Behnood et al. [43] reviewed and compared the methods for monitoring pH in concrete and divided them into destructive and non-destructive methods. Destructive methods are the most used for concrete condition assessment. However, these are limited by sampling, which may not be representative of the whole structure [44]. Behnood et al. described the three most used destructive methods for pH monitoring; namely: the pore water expression method, that extracts the concrete pore solution under hydraulic pressure; the in-situ leaching method, which analyses the concrete pore solution inside a cavity by equilibrium with an added solution; and the ex-situ leaching method, that is based on a powder solution and by equilibration with an added solution. Pore water expression is the most used from the three methods above, and it was recommended by Plusquelle et al. [45] mainly due to its simplicity. On the other hand, non-destructive methods, which are becoming more and more evolved, can be electrochemical or optical [44]. These monitoring methods started with the development of electrochemical sensors for the measurement of corrosion potential of rebars [44]. However, in the last few years, there has been a huge interest in the development of new optical sensors which are generally based on properties such as fluorescence, absorbance, reflectance and refractive index [46]. Optical fibre sensors (OFS) for applications in concrete structures show several advantages when compared to electrochemical methods, such as reduced cost, size and weight, as well as higher sensitivity [39]. Behnood et al. concluded that OFS can be very effective for real-time pH monitoring. Nevertheless, the sensors already reported for application in concrete need to be further developed in order to obtain accurate levels of resolution, repeatability and reproducibility [43].

The use of OFS for concrete monitoring was introduced long ago, mainly for crack monitoring [47] and other mechanical variations. Since then, OFS based on sol-gel materials have been developed for structural health monitoring [39,43,45,48,49,50], including parameters such as temperature, humidity and pH [39]. However, most of the developed OFS functionalized with OIH sol-gel films have limitations for concrete applications, such as the leaching of the doped species from the films or poor resistance to be used in fresh concrete. As far as the authors know, no OFS functionalized with PDMS-based OIH sol-gel films for concrete pH monitoring have been described. PDMS hydroxyl-terminated has been reported as a concrete additive [51] to reduce the production of calcite protecting the concrete structures against the reduction of the pH level. Sidek et al. [52,53] reported the preparation of a fibre Bragg grating (FBG) sensor for strain detection in concrete structures, with and without a PDMS coating. PDMS coated FBG sensors have shown themselves to be more sensitive than uncoated FBG sensors [54]. Later, in 2016, Tan et al. [55] showed that PDMS-coated FBG indeed enhanced the sensibility of strain detection and that the response of the sensor was linear to the rebar corrosion rate, estimated by the weight loss. PDMS has also some limitations, such as its low Young’s modulus and the possibility to absorb impurities during the curing process [55]. Nevertheless, the combination of the OFS technology and PDMS-based OIH show the potential to build a non-destructive monitoring system that can withstand the harsh conditions of fresh concrete. Phenolphthalein (Phph) is one of the most well-known pH indicators and has its turning point at a pH between 8 and 10. This indicator is already used for qualitative assessment of the depth of carbonation in concrete (e.g., by staining concrete cores right after extraction from a given structure) [56]. Therefore, OIH sol-gel materials doped with Phph can be used to functionalise OFS, allowing one to assess the carbonation of concrete structures.

This work reports a study that is only focused on the synthesis and characterization of three OIH sol-gel materials based on PDMS-diglycidyl, ether terminated (PDMS(800)-GET). The chemical characterization and stability testing of the OIH matrices in contact with simulating concrete pore solution (SCPS) were carried out. The development of OFS based on OIH sol-gel materials opens up the possibility of producing highly accurate and reliable sensing systems. Therefore, the aim of these studies was to assess the ability of these materials to be used as sensing material for OFS functionalisation. The produced films, doped and undoped with Phph, were characterized by Fourier-transformed infrared (FTIR) spectroscopy, UV-Vis spectroscopy, electrochemical impedance spectroscopy (EIS) and thermogravimetric analysis (TGA). UV–Vis characterization confirmed the presence of Phph embedded in the OIH matrices. The dielectric and thermal properties of the materials showed promising properties for application in contact with a high alkaline environment.

## 2. Materials and Methods

### 2.1. Materials

Poly(dimethylsiloxane)-diglycidyl ether (PDMS(800)-GET, Sigma-Aldrich, St. Louis, MO, USA), 3-aminopropyltriethoxysilane (3-APTES, 99%, Acros Organics, Geel, Belgium), phenolphthalein (Merck, Darmstadt, Germany), absolute ethanol (EtOH, PanReac, Darmstadt, Germany), citric acid monohydrate (Merck, Darmstadt, Germany), potassium bromide (KBr, FTIR grade, ≥99%, Sigma-Aldrich, St. Louis, MO, USA), calcium hydroxide (Ca(OH)_2_, 95%, Riedel, Bucharest, Romania) and potassium hydroxide (KOH, 90%, PanReac, Darmstadt, Germany) were used as received. High purity deionized water with high resistivity (higher than 18 MΩ cm) obtained from a Millipore water purification system was used in all prepared solutions.

### 2.2. Synthesis of Organic-Inorganic Hybrid (OIH) Films

#### 2.2.1. General Procedure

**Figure 1:**
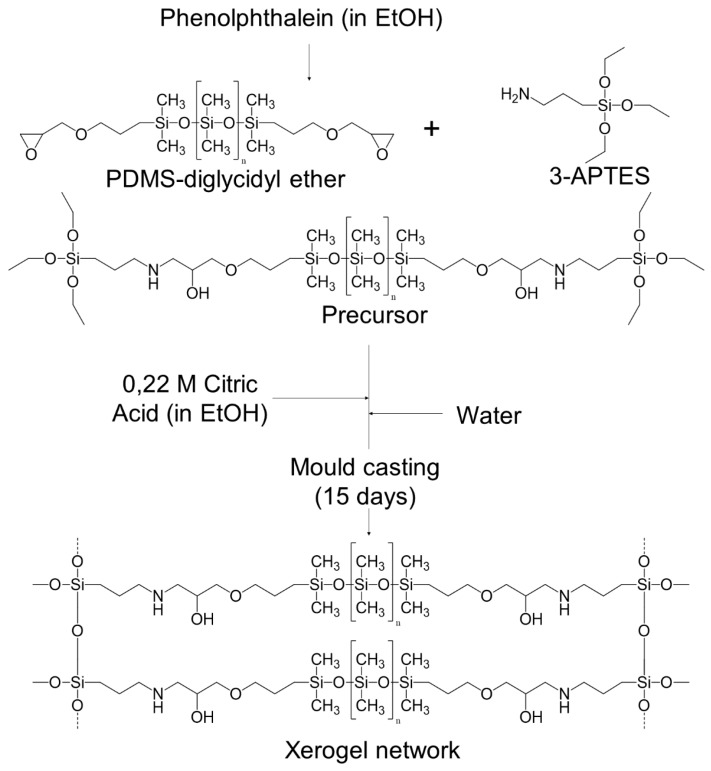
Schematic of the main steps used in the synthesis of PDMS based OIH films.

The first stage of the hybrid films’ synthesis consisted of the formation of a covalent bond between PDMS(800)-GET and 3-APTES (Table 1, Figure 1). Different ratios between the two precursors were used (1:2, 1:2.5 and 1:5 PDMS: 3-APTES). The two precursors were mixed in a glass container for 20 min. A 0.22 M citric acid ethanolic solution was added to set a ratio of 0.094 between citric acid and the corresponding amount of silane. The mixture was stirred for another 20 min and water was added to set a H_2_O/PDMS ratio of 29.7. The solution was stirred for more 20 min to obtain a homogeneous mixture and cast into a Teflon^®^ mould. The Teflon^®^ mould was sealed with parafilm^®^ and placed in a universal oven (UNB 200, Memmert, Buechenbach, Germany) and kept at 40 °C for 15 days in order to ensure the curing of the films and evaporation of the remaining solvents. Films were identified as AES(800)-1/2, AES(800)-1/2.5 and AES(800)-1/5.

#### 2.2.2. Phph Doped Films

PDMS(800)-GET (1 mmol) was stirred with a 0.1 M ethanolic solution of Phph, according to Table 1, for 20 min in a glass container; 3-APTES (2 mmol, 1/2 PDMS/3-APTES ratio) was added to the solution and the general procedure was followed. However, in this particular case water was not added into the solution. Films were identified as AES(800)-1/2-Phph-0.01, AES(800)-1/2-Phph-0.02 and AES(800)-1/2-Phph-0.05.

### 2.3. Characterization of the OIH Films

The OIH pure films were characterized by Fourier-transformed infrared (FTIR) spectroscopy, UV-Vis spectroscopy, electrochemical impedance spectroscopy (EIS) and thermogravimetric analysis (TGA). The three OIH films doped with Phph were only characterized by UV-Vis and EIS.

#### 2.3.1. FTIR

FTIR spectra for AES(800)-1/2 were recorded in transmittance mode on a Bomem MB104 spectrometer, by averaging 20 scans at a maximum resolution of 4 cm^−1^. Spectra were obtained in 4000-700 cm^−1^ range on KBr pellets. For the analysis during synthesis, a 2 µL droplet of the solution was placed in the previously prepared 100 mg KBr pellet. Droplets were collected 20 min after both precursors were added, before hydrolysis and condensation of amino alcohol precursor. Liquid precursors KBr pellets followed the same preparation. For the final OIH films, KBr pellets were prepared with 0.1 mg of cured material and 200 mg of KBr.

#### 2.3.2. UV–Vis Spectroscopy

UV–Vis spectra for both pure and doped OIH films were recorded in absorbance mode on a Shimadzu UV-2501 PC spectrophotometer. Spectra were obtained in the range of 200–800 nm for solid samples.

#### 2.3.3. Electrochemical Impedance Spectroscopy (EIS)

EIS measurements were carried out on all the produced OIH disc materials at room temperature in a Faraday cage, using a potentiostat/galvanostat/ZRA (Reference 600+, Gamry Instruments, Warminster, PA, USA). EIS measurements were used to characterize resistance, electrical conductivity and electric permittivity of the prepared OIH disc materials. The capacitance of OIH films was also determined. Measurements were performed using a support cell as reported in previous studies [57]. The disc films were placed between two parallel Au electrodes (10 mm diameter and 250 μm thickness) using the mentioned support cell and the EIS measurements were conducted [57]. EIS studies were accomplished by applying a 10 mV (peak-to-peak, sinusoidal) electrical potential within a frequency range from 1 × 10^6^ Hz to 0.01 Hz (10 points per decade) at open circuit potential (OCP). The frequency response data of the studied electrochemical cells were displayed in a Nyquist plot, using Gamry ESA410 Data Acquisition software that was also used for data fitting purposes. For chemical stability studies in an alkaline environment, the OIH discs were immersed in a simulative concrete pore solution (SCPS) with a pH of 13.5. The SCPS was prepared, using deionized water at room temperature according to M. Sanchez et al. [58] and F. J. Recio et al. [59]. Generically, the SCPS was obtained by addition of 0.2 M KOH to a Ca(OH)_2_ saturated solution [58,59].

#### 2.3.4. TGA

Thermal analysis was carried out on an SDT Q600 system for the three pure OIH materials. Samples were subjected to a temperature ramp of 10 °C/min between room temperature and 800 °C at a constant 40 mL/min nitrogen flux. For each analysis, 15 mg of each OIH material was placed into an alumina pan.

## 3. Results and Discussion

### 3.1. FTIR Analysis

**Figure 2:**
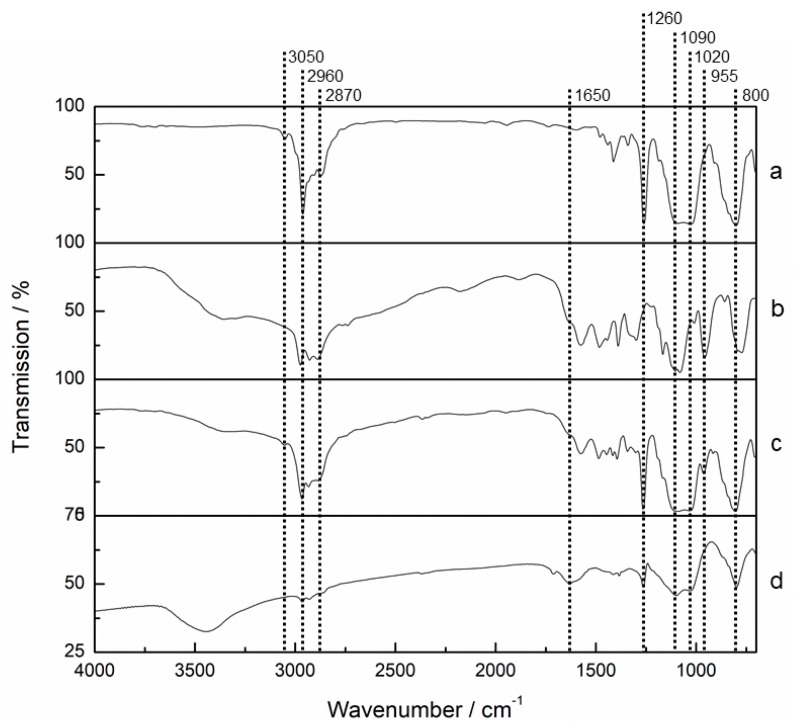
FTIR transmittance spectra of AES(800)-1/2. (**a**) PDMS(800)-GET; (**b**) 3-APTES; (**c**) AES(800)-1/2 during synthesis; (**d**) AES(800)-1/2 final hybrid (presented with a different scale, for higher definition purposes).

FTIR analysis was carried out for AES(800)-1/2 and is shown in Figure 2. Spectra of both precursors ((a) and (b)), from a sample during synthesis after 20 min of precursors addition (e.g., before hydrolysis and condensation of amino alcohol precursor) (c) and the final OIH material (d) are depicted.

PDMS(800)-GET spectrum (a) from Figure 2 shows a weak band at 3050 cm^−1^, corresponding to the epoxy terminal group [60]. Bands between 2960 and 2870 cm^−1^ may be ascribed to C-CH_2_ asymmetric and symmetric stretching vibrations. A well-defined sharp peak at 1260 cm^−1^ belonging to C–Si bond symmetric bending is especially characteristic of dimethylsiloxane chains [61]. Broad bands at 1090 and 1020 cm^−1^ are related to Si–O–C and Si–O–Si bonds and the peak at 800 cm^−1^ corresponds to C–Si bond symmetric rocking vibration [61,62]. 3-APTES spectrum (b) from Figure 2. also shows the bands corresponding to C–CH_2_ asymmetric and symmetric stretching vibrations. Besides, a broad band between 3500–3300 cm^−1^ may be ascribed to N–H bond stretching vibrations of the terminal amine group involved in H-bonds. Peaks at 1100, 1080 and 955 cm^−1^ are characteristic of Si–O–CH_2_CH_3_ bonds of this precursor [61]. In the spectrum of the hybrid film during its synthesis (c) from Figure 2, the epoxy group peak present in PDMS spectra (3050 cm^−1^) disappeared, as expected, due to the reaction between this group and the amine group from the silane. The broad band between 3500–3300 cm^−1^ is also present, showing no difference between primary (3-APTES) and secondary (hybrid films) amines. The peak corresponding to C–Si symmetric bending (1260 cm^−1^) is still present, as well as the peaks between 2960 and 2870 cm^−1^ belonging to C–CH_2_ asymmetric and symmetric stretching vibrations and the peaks at 1090, 1020 and 800 cm^−1^, from the dimethylsiloxane chain. The Si–O–CH_2_CH_3_ characteristic peak (955 cm^−1^) is still present but on a minor scale. Final hybrid film spectrum Figure 2. (d) does not show this peak, due to the total hydrolysis of these alkoxy groups. A small peak at 1650 cm^−1^ appears, which may belong to the C–NH–C bond bending vibration, corresponding to the formed amino alcohol bond. The broad and intense band at about 3450 cm^−1^ can be related not only to the hydroxyl group formed in the film but also to water molecules that remained entrapped in the polymeric matrix.

### 3.2. UV–Vis Analysis

**Figure 3:**
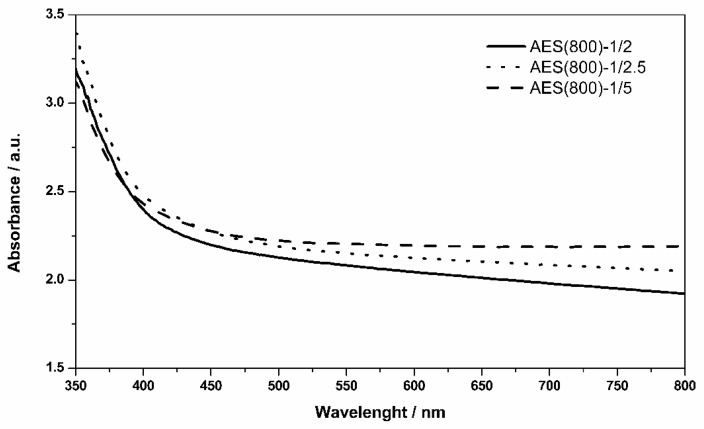
Absorption spectra of pure hybrid samples.

Optical absorption spectra for the three pure OIH samples are shown in Figure 3. Even though the samples are not transparent, absorbance in the range of 400–800 nm is constant, showing the films can be doped with a colorimetric chemosensor that absorbs in this range.

**Figure 4:**
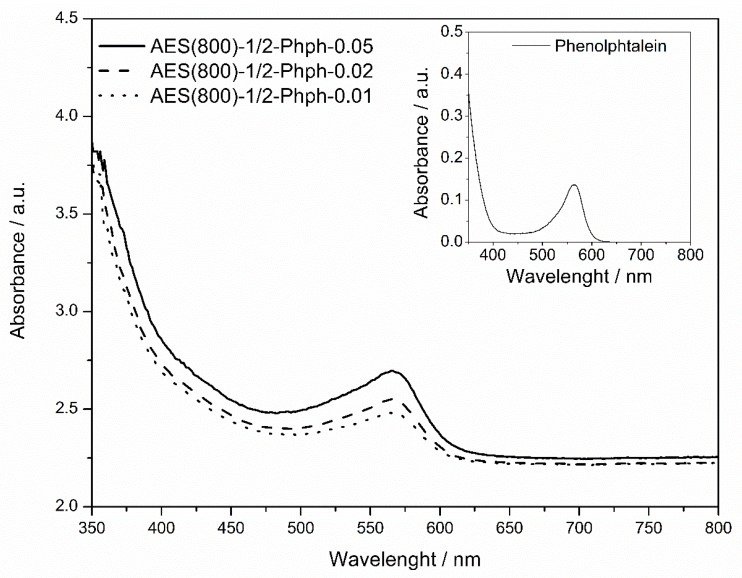
Absorption spectra of AES based FFT doped films. Inset: absorption spectrum of the basic form of Phph in ethanol.

Phph, a well-known pH indicator, colourless below pH 8 and pink above pH 10, was immobilized in the sol-gel matrix. UV–Vis spectroscopy analysis was carried out to confirm the presence of the indicator in the matrix. The spectra for AES based Phph doped films are shown in Figure 4. The absorption spectrum of the basic form of Phph in ethanol is also shown as inset. The three spectra show a peak around 566 nm, corresponding to the basic form of Phph, confirming its presence within the OIH material. Since the Phph peak in ethanol and within the matrix share the same wavelength, it can be assumed that the molecule does not change the electron distribution profile that is involved in the radiation absorption.

The results show (Table 2) that the absorption coefficients for Phph on the three doped films are of the same magnitude order. That result was expected, since the matrix has a similar composition. Distinctively from the ethanol solution, the molecule entrapped within a rigid matrix contributes to molecule–matrix interaction, reducing the efficiency of the radiation absorption.

### 3.3. EIS Analysis

**Figure 5:**
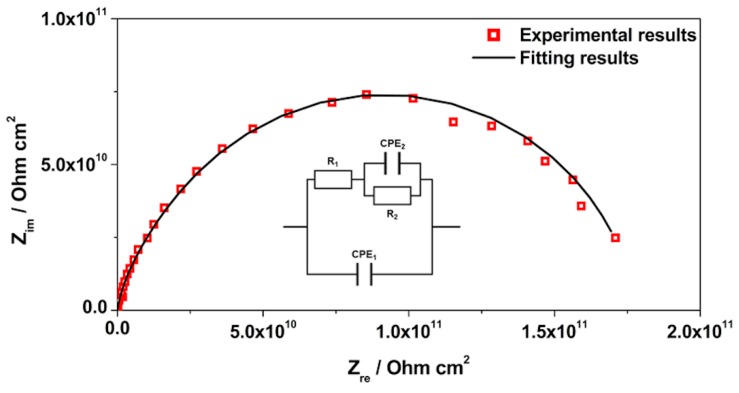
Nyquist plot for pure OIH AES(800)-1/2.

**Figure 6:**
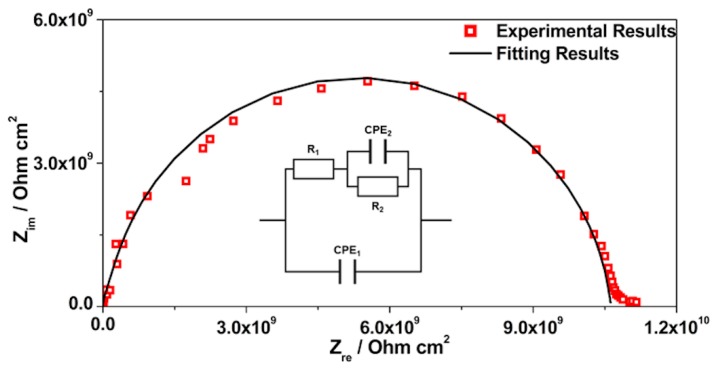
Nyquist plot for pure OIH AES(800)-1/2.5.

**Figure 7:**
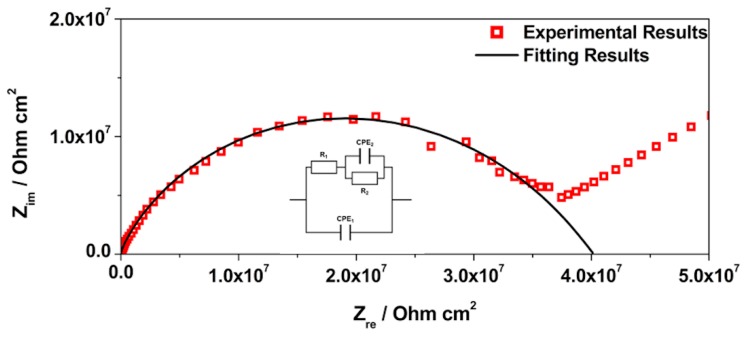
Nyquist plot for pure OIH AES(800)-1/5.

**Figure 8:**
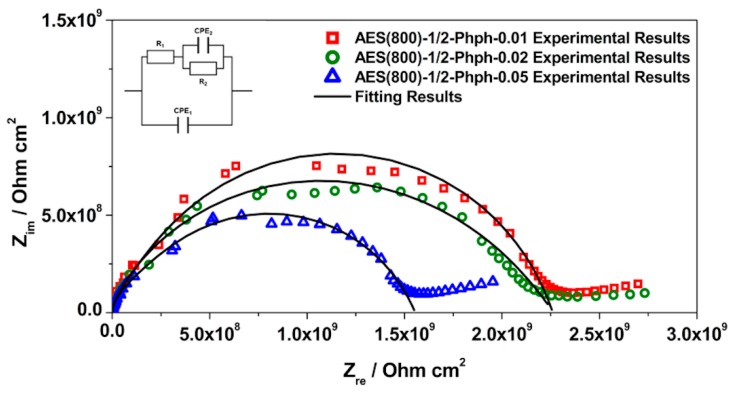
Nyquist plot for Phph doped OIH AES(800)-1/2-Phph films.

EIS is a technique widely used for materials characterization. EIS allows one to obtain the dielectric properties of the OIH films and can be extremely helpful on optimizing the OIH materials in the synthesis procedure. Considering the information that can be extracted (e.g., capacitance, resistivity, dielectric permittivity, etc.) from EIS measurements, it is possible to follow the degradation of the OIH materials by quantifying the changes in the dielectric properties [63,64]. When EIS is performed on high performance materials, the Nyquist plots show a typical capacitive response over a wide range of frequencies. In the high-frequency region of the Nyquist plots, the dielectric properties of the material can be extracted. In this work, the EIS was used to characterize the dielectric properties (e.g., conductivity, capacitance and electric permittivity), which can be linked to the degradation properties of the material. Therefore, these studies allow one to assess the potential of the OIH matrices to be used in contact with a high alkaline environment (fresh concrete). Figure 5, Figure 6 and Figure 7 show the Nyquist plots of the pure OIH materials identified according to Table 1. Figure 8 shows the Nyquist plots of the OIH materials doped with Phph, which were also identified according to Table 1. The fitting results are also displayed in all the Nyquist plots. The equivalent electrical circuit (EEC) to describe the impedance spectra response was also included in each figure.

Figure 5, Figure 6 and Figure 7 show that at higher frequencies both Nyquist plots describe a semicircle, with the exception of the AES(800)-1/2.5 sample (Figure 6), which intersects the x-axis; none of the other samples displayed this behaviour. The amplitudes of the different samples change with their composition, and the undoped samples show the higher impedance magnitudes. The AES(800)-1/2 sample (Figure 5) showed the highest magnitude, and was followed by the AES(800)-1/2.5 (Figure 6). Sample AES(800)-1/5 showed the lowest magnitude. These differences are assigned to the dielectric properties of the OIH materials (e.g., resistivity and capacitance). The data obtained at lower frequencies describes a line in AES(800)-1/5 (Figure 5). The same behaviour was found in all OIH materials doped with Phph. This suggests that another electrochemical process, which is attributed to the Au|OIH material interface, is taking place. This part of the Nyquist plot describes a simple parallel resistance and capacitor EEC; however, the fitting was disregarded, since it is not relevant for the comprehension of the dielectric properties of the OIH materials. The analysis of EIS data was based on the represented EEC. Constant phase elements (CPE) were used instead of pure capacitance, since the results obtained (Figure 5, Figure 6, Figure 7 and Figure 8) do not show ideal behaviour. The impedance of a CPE can be defined as [65]:

Z_CPE_ = 1/[Q(jω)^α^]. (1)

When α = 1, Q represents the capacity, and if α ≠ 1, the system shows a behaviour that is linked to the surface heterogeneity. If the system shows this second behaviour instead of an ideal capacitor, the impedance for the EEC is determined by [66]:

Z_CPE_ = R_Sample_/[1 + (jω)^α^QR_Sample_], (2)

where R_Sample_ is the resistance in parallel with the CPE. The CPE parameter Q cannot be considered as the interfacial capacitance (C_eff_). C_eff_ is determined by [66,67]:

C_eff_ = [QR_sample_^(1−α)^]^1/α^. (3)

The EEC used to fit the Nyquist plots obtained for all samples contains two CPEs (CPE_1_ and CPE_2_) and two resistances (R_1_ and R_2_) which are related to the resistance of the OIH material. The observation of the Nyquist plots (Figure 5, Figure 6, Figure 7 and Figure 8) shows, at higher frequencies, two partially overlapping semicircles with different radii. This behaviour may be identified as two time-dependent charge relaxation processes with two different time-constants (CPE_1_ and CPE_2_). The systems show a frequency dispersion linked to the relaxation phenomena that may be explained by the presence of residual solvent incorporated within the OIH matrix. The elements R_1_ and R_2_ depict the sample’s (bulk) resistance of these two distinct dielectric media and according to this EEC, the sample’s bulk resistance is the sum of R_1_ and R_2_. Three measurements were performed for each sample. However, only representative values together with the respective fitting parameters and the percentage of error associated with each element parameter are shown in Table 2.

Table 3 shows that all the resistances are within the range of 10^7^–10^11^ Ω cm^2^. This suggests that the studied OIH materials display suitable resistance values which may imply that the synthesized materials can endeavour the harsh conditions of the fresh concrete [68]. The electrical resistance of the OIH AES(800)-1/2 is the highest, and the resistance of the OIH AES(800)-1/5 is the lowest (Table 2). This is in accordance with the literature [7,68], since the higher the ratio between the organic and inorganic amounts of precursors, the lowest the resistance. Table 2 also shows that the OIH materials doped with Phph are of the same magnitude order (10^9^). Moreover, the samples AES(800)-1/2-Phph-0.01 and AES-1/2Phph-0.02 have equal electrical resistance values. This behaviour is expected, since the ratio between PDMS and 3-APTES is the same for the OIH materials doped with Phph.

**Figure 9:**
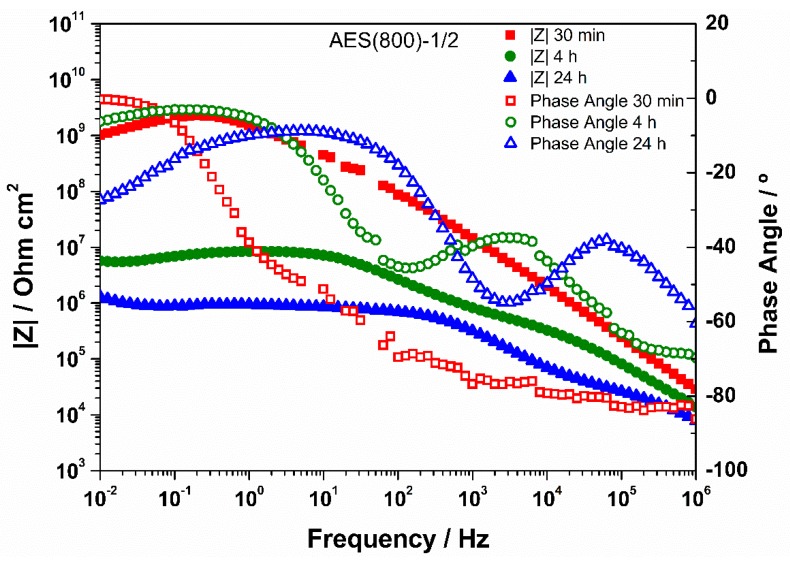
Bode plot for EIS analysis of AES(800)-1/2 after 30 min, 4 h and 24 h in SCPS.

**Figure 10:**
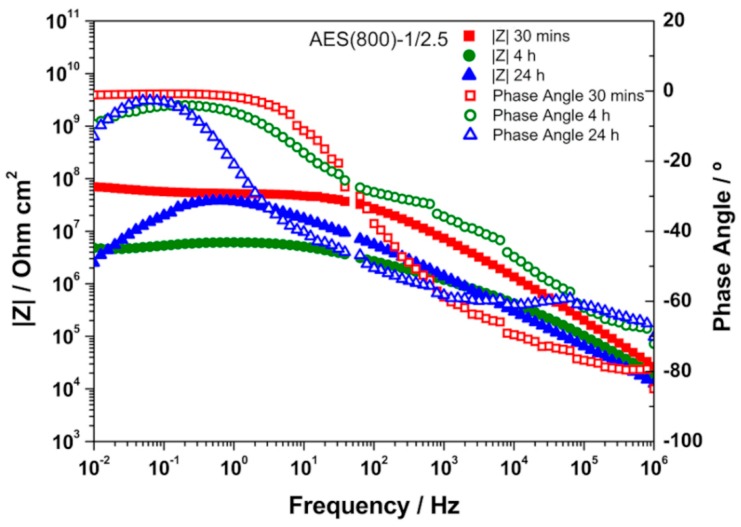
Bode plot for EIS analysis of AES(800)-1/2.5 after 30 min, 4 h and 24 h in SCPS.

**Figure 11:**
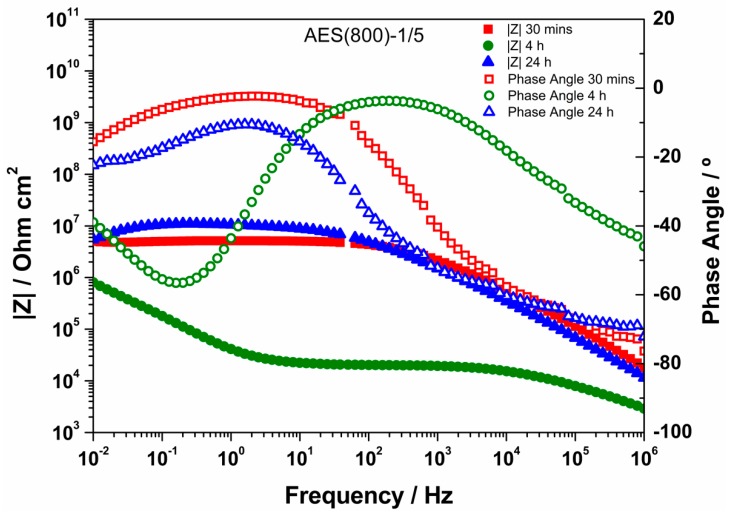
Bode plot for EIS analysis of AES(800)-1/5 after 30 min, 4 h and 24 h in SCPS.

To assess the chemical stability and resistance of the pure OIH materials, these were immersed in SCPS, which was prepared as described in Section 2.3.3. This solution was used since it is generally accepted as the most representative of concrete pore solution [58,59], because it simulates the alkalinity and the high alkali content of the pore solution existent in the concrete structures. The EIS measurements of the OIH films in contact with SCPS were conducted after 30 min, 4 h and 24 h of immersion. The Bode plots obtained are shown in Figure 9, Figure 10 and Figure 11.

High impedance modulus |Z| at low-frequency (LF) ranges indicate the level of porosity and defects present in the OIH films [69]. Generally, for all OIH samples, as the immersion time increases the impedance modulus decreases. For OIH samples AES(800)-1/2, Figure 9 shows that this value decreases as the immersion time in SCPS increases. The highest decrease (about two magnitude orders when compared to samples immersed for 30 min) was found after 4 h of immersion. After 24 h, the decrease is lower than one order of magnitude when compared to samples after 4 h of immersion. For OHI samples AES(800)-1/2.5 (Figure 10) the bode plots are similar after 4 h and 24 h, but decreased about one order of magnitude when compared to samples immersed for 30 min. For AES(800)-1/5 samples, shown in Figure 11, there is a clear decrease after 4 h of immersion. However, after 24 h of immersion, the impedance shows a similar behaviour when compared to samples immersed for 30 min. This may be explained by the lower ratio between the organic and inorganic amounts of precursors.

Generally, the results show that the dielectric properties of the OIH materials change during the immersion in SCPS (pH > 12.5). Nevertheless, the |Z| values are within the range of 10^6^–10^9^. This behaviour suggests that the OIH materials synthesized show suitable properties to be embedded in fresh concrete. Furthermore, according to previous studies [68] materials with similar values were able to resist to the high pH of mortars.

### 3.4. TGA Analysis

**Figure 12:**
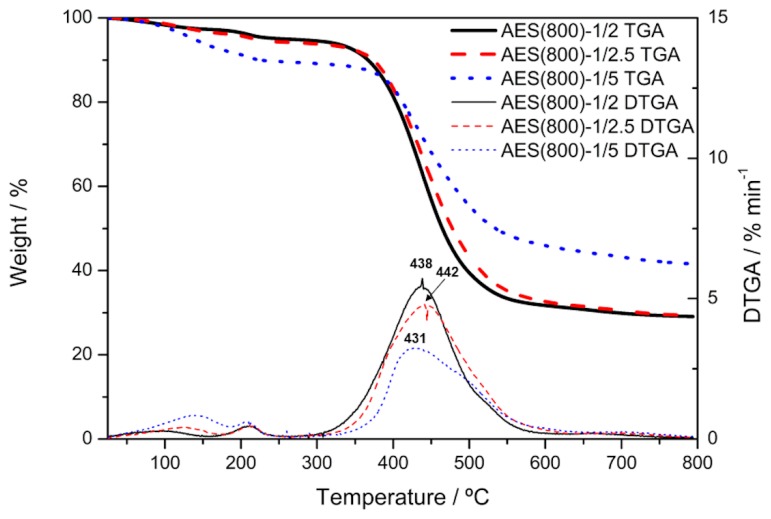
Experimental TGA and DTGA traces for AES(800)-1/2, AES(800)-1/2.5 and AES(800)-1/5 obtained at a heating rate of 10 °C/min.

Figure 12 shows that the highest degradation processes of the OIH materials occurred in the range between 400 and 600 °C, which is according to the literature [70,71,72,73]. Higher weight loss is shown for the sample with a smaller 3-APTES/PDMS ratio. These degradation processes are due to the depolymerization of the matrices and cleavage of Si–C bonds. The sample with the higher 3-APTES/PDMS ratio (AES(800)-1/5) has a smaller percentage of weight loss due to depolymerization. This may be explained by the decrease in the ratio between the organic and inorganic amounts of precursors. Table 3 shows the 5% weight loss temperature (T_5_) and the temperature of the maximum rate of weight loss (T_max_). It is shown that the higher the stoichiometric molar ratio between 3-ATPES and PDMS, the lower is the T_5_ due to the higher volatile content. T_max_ (Table 4) does not show a clear association with this ratio. However, the maximum rate of weight loss is higher for smaller ratios between organic and inorganic amounts of precursors.

The TGA/DTGA data show that the main degradation processes occurred at temperatures above 350 °C; therefore, these OIH materials are suitable to apply in fresh concrete, since the maximum values achieved during the concrete curing process reach temperatures around 70 °C [74].

## 4. Conclusions

New OIH sol-gel materials based on PDMS(800)-GET and 3-APTES, with different ratios, were synthesized and were characterized by FTIR, UV–Vis spectroscopy, EIS and TGA. Phph-doped OIH films were also synthesized. FTIR spectroscopy showed that the synthesis of the precursor by the reaction between PDMS(800)-GET and 3-APTES was successful by the disappearance of the PDMS-characteristic epoxy group band, as were the hydrolysis and condensation of this precursor, as shown by the disappearance of alkoxy group’s band. The presence of the indicator on the doped films was confirmed by UV–Vis spectroscopy.

Dielectric and thermal properties of the materials show that these materials resist high alkaline environments (SCPS, pH > 12.5). The impedance values allow one to conclude that for the OIH films, after immersion in SCPS, they do not decrease significantly. TGA data show that the produced OIH materials are stable enough to be used in fresh concrete, since the thermal degradation only occurs for values above the curing process of concrete (70 °C). The results indicate that these materials have potential to be used as sensing material and functionalize OFS to assess the pH of concrete. However, further studies must be conducted on functionalized OFS in SCPS in order to validate the potentiality of these OIH materials on OFS.

## Figures and Tables

**Table 1:** Adopted codes for organic-inorganic hybrid (OIH) films.

OIH Films	PDMS:APTES Ratio	Phph:PDMS Ratio
AES(800)-1/2	1:2	-
AES(800)-1/2.5	1:2.5	-
AES(800)-1/5	1:5	-
AES(800)-1/2-Phph-0.01	1:2	1:100
AES(800)-1/2-Phph-0.02	1:2	1:50
AES(800)-1/2-Phph-0.05	1:2	1:20

**Table 2:** UV–Vis data values obtained from the doped OIH materials spectra (Figure 4).

Phph	Ethanol	AES(800)-1/2-Phph-0.01	AES(800)-1/2-Phph-0.02	AES(800)-1/2-Phph-0.05
Absorbance (566 nm) *	0.136	0.411	0.482	0.627
[Phph]/mol g^−1^	1.27 × 10^−7^	8.05 × 10^−6^	1.48 × 10^−5^	2.62 × 10^−5^
Optical path/cm	1.000	1.131 **	0.919 **	1.017 **
Absorption coefficient/10^4^ g mol^−1^ cm^−1^	107.08	4.52	3.55	2.35

* For the OIH films, the absorbance of the corresponding OIH undoped (AES(800)-1/2) was subtracted. ** Thickness of the film.

**Table 3:** EEC data parameters values obtained by fitting the EIS response obtained for both doped and undoped films (Figure 5, Figure 6, Figure 7 and Figure 8).

Sample	CPE_1_/S^α^ Ω^−1^ cm^−2^	α_1_	R_1_/Ω cm^2^	CPE_2_/S^α^ Ω^−1^ cm^−2^	α_2_	R_2_/Ω cm^2^	R_sample_/Ω cm^2^	χ^2^
AES(800)-1/2	9.27 × 10^−12^	0.900	9.00 × 10^10^	8.12 × 10^−12^	0.900	8.84 × 10^10^	1.78 × 10^11^	2.24 × 10^−1^
	(1.54%)	(0.25%)	(0.01%)	(2.75%)	(0.51%)	(2.20%)		
AES(800)-1/2.5	6.45 × 10^−12^	0.975	1.47 × 10^9^	5.35 × 10^−12^	0.881	9.16 × 10^9^	1.06 × 10^10^	4.81 × 10^−3^
	(2.17%)	(0.19%)	(13.10%)	(8.36%)	(2.90%)	(2.61%)		
AES(800)-1/5	2.46 × 10^−11^	0.910	2.10 × 10^6^	5.28 × 10^−10^	0.575	3.81 × 10^7^	4.02 × 10^7^	3.58 × 10^−4^
	(±13.19%)	(±0.97%)	(±9.90%)	(±10.64%)	(±2.57%)	(±3.00%)		
AES(800)-1/2-Phph-0.01	6.60 × 10^−12^	0.961	3.17 × 10^8^	2.97 × 10^−11^	0.748	1.94 × 10^9^	2.26 × 10^9^	2.61 × 10^−3^
	(±2.73%)	(±0.23%)	(±7.96%)	(±7.11%)	(±2.56%)	(±2.24%)		
AES(800)-1/2-Phph-0.02	7.61 × 10^−12^	0.964	1.94 × 10^8^	6.47 × 10^−10^	0.597	2.07 × 10^9^	2.26 × 10^9^	1.88 × 10^−3^
	(±3.26%)	(±0.27%)	(±15.50%)	(±5.95%)	(±3.01%)	(±2.63%)		
AES(800)-1/2-Phph-0.05	8.32 × 10^−12^	0.954	2.82 × 10^8^	5.99 × 10^−11^	0.713	1.27 × 10^9^	1.55 × 10^9^	1.31 × 10^−3^
	(±2.61%)	(±0.00%)	(±7.40%)	(±7.96%)	(±3.34%)	(±3.01%)		

**Table 4:** Data collected from TGA and DTGA traces.

Films	T_5_	T_max_
AES(800)-1/2	256	438
AES(800)-1/2.5	213	442
AES(800)-1/5	135	431
